# Feeling safe together vs. longing for touch. Affective, multisensorial contact in inclusive intra-active virtual/physical dance during the COVID-19 pandemic

**DOI:** 10.3389/fpsyg.2024.1325982

**Published:** 2024-02-19

**Authors:** Noora Talvikki Oertel

**Affiliations:** Doctoral Programme in Gender, Culture and Society, Faculty of Arts, University of Helsinki, Helsinki, Finland

**Keywords:** COVID-19, inclusive dance, touch, virtual/physical dance, feelings of safety, haptic visuality, corpomateriality, affect

## Abstract

This article explores how feelings of safety were experienced through inclusive virtual/physical dance in relation to experiences of touch during the COVID-19 pandemic in Finland. The following are the measures I took to achieve the aim of this study. First, I introduce the context through previous studies and an example from the ethnographic material I gained from the inclusive X-Dance festival organized in June 2021 in Finland. Second, I explore how inclusive virtual/physical dance might help us experience feelings of safety through multisensorial experiences of touch related to communality when responding to wellbeing challenges caused by isolation. Using Laura U. Mark's theory about haptic looking and Karen Barad's term intra-action, I discuss different possibilities to approach touch as a feeling sense, as affective multisensorial contact, and through relations between different materialities. Third, I contrast these ideas with sensations of longing for the physical touch that virtual/physical dancing evokes. These experiences of longing invite me to reflect on the ambivalence about feelings of safety related to intentions to restore experiences of touch and communality through digitality. I introduce two examples from my interviews with dancers during the pandemic to discuss these ambivalences. I reflect on these interviews through Magdalena Górska's theory about corpo-materiality and corpo-affectivity. I contribute to discussions on feelings of safety by showing that multi-sensorial experiences and anti-normative understandings of body and touch enabled by the non-verbal language of dance may help us to contribute toward more inclusiveness in society, allowing us to generate holistic experiences of safety, which is another critical aim for post-pandemic times.

## 1 Introduction

During the COVID-19 pandemic, different online practices replaced physical meetings to enable interaction, socialization, and work. The new forms of interaction recommended by governments and health authorities were intended to increase safety in society by preventing the virus from spreading. However, the different measurements taken affected some people's feelings of safety in contradictory ways. For example, psychological distress, anxiety, and depression were experienced due to isolation and the lack of physical touch (Lakhan et al., 2020; Simşir et al., 2022).

Touch is one of the senses that provide feelings of safety for some individuals. Touch connects us to the environment and our bodies. Ironically, the different safety measures led to the loss of touch and, thus, the loss of safety, affecting people's wellbeing, especially those living alone (Field et al., 2020, p. 12; Hopf et al., 2022). For example, the elderly and people with disabilities risk being grievously affected by a potential infection and are excluded from the digital replacements of socializing and (health) care (Cho and Kim, 2022; Heponiemi et al., 2022; Zapletal et al., 2023).

Different artistic online practices aimed to restore the feelings of safety by inventing alternative ways to connect and touch. In this article, I discuss how feelings of safety are experienced regarding different tactile experiences through inclusive virtual/physical dance^1^. I draw on an example from my attendance at the inclusive (physically integrated dance/ability dance)^2^ X-Dance festival organized by DanceAbility Finland and the KAAOS company in June 2021. I also discuss two examples of 11 interviews that I conducted in 2021 with dancers from different organizations who practice inclusive dance.

The bodily perspectives I take in this article through inclusive dance help me approach the feelings of safety regarding the anti-normative, situated understandings about the body and touch. This is important when responding to the social inequalities that push some people into marginalized and vulnerable positions that tend to increase in times of crisis (Siller and Aydin, 2022). This article is written under the influence of the disability culture movement that has the wider aim of seeking ways of undoing the history of exclusions relating to internalized ableism, racial stereotypes, class, gender, and economic access (Kuppers, 2011, p. 4). I engage with affect theories influenced by new materialist approaches to challenge the normativity of the body and touch.

## 2 Relations between feelings of safety, affect, and emotions

Crises such as COVID-19 tend to affect our experiences of identity and the body through how they regulate our movements and influence our feelings and social interactions. I understand feelings as subjective, phenomenological experiences (Blackman and Cromby, 2007, p. 5–6), while I refer to affect as bodily or sensorial experienced processes of becoming (Massumi, 1995, p. 88). These processes can be experienced in situations where the subjects affect and become affected by other living beings, things, situations, material, discourses, etc. (Braidotti, 2002; Latour, 2004; Rinne et al., 2020).

According to cultural and media theorist Lisa Blackman and psychologist John Cromby “[...] feelings register intensive forces [affect] as subjective experience, and emotions performatively stabilize these forces into culturally normative patterns through practices of expression, movement, or speech” (Blackman and Cromby, 2007, p. 6). Thus, when I write about feelings of safety, I refer to individual experiences influenced by affect. Sometimes, these feelings can turn into culturally shared, performative emotions (Rinne et al., 2020, p. 8).

Due to the processual, fluid, and moving character of affect, they can destabilize normative ideas of the body, hence challenging the organization of body politics. Dance artist and cultural theorist Erin Manning writes, how thinking about the body in relation to the senses encourages us to approach the body in movement, which enables us, for example, to “engage with the possibility that bodies are not limited to their organs” (Manning, 2006, p. xv) and to focus on “what the body can do” instead of reproducing essentialist ideas about “what the body is” (Manning, 2006, p. xv).

The perspective on the role of affect, movement, and touch in experiences of embodiment makes me approach feelings of safety through different affective intra-active relations (Barad, 2007). I use the concept of intra-action, introduced by feminist philosopher and physicist Karen Barad, to highlight the relational character and the “in-betweenness” of different situations of exchange and affection between different materialities (ibid., 141), such as screens, cameras, the COVID-19 virus, and humans. Furthermore, I discuss how safety can be individually felt due to these affective relations.

## 3 Feeling safe together vs. longing for physical touch

*We are about eight dancers, moving forward and backward in front of the camera and from one side to the other. Parts of the dancers' bodies come close, I would like to say to my skin, but I have to restrict my perception to my eyes. In another moment, the dancers disappear completely from the picture. We take impulses from each other. Sometimes the focus is on our hands and fingers, then it switches to the head that moves as a protagonist. A dancer lifts her feet and starts a funny quintet of her five toes. Two other dancers join the movement series of the dancing toes while another dancer starts a fluid, wavy movement*.

*We exchange movement qualities in different duets, trios, quartets, and solos, happening parallel with the rhythms and melodies of the music. I feel moved by the connection between the different bodies. Even though I do not know the other participants, there is a connection between us. After the class, I feel a teardrop running along my cheek, the taste of salt touching my lips. Getting in touch, being touched is out of control [sic]* (Morning class, XDF 6/6/2021).

The physical/virtual dance class, which I presented in the note above, enabled me to diversify my understanding of my body when I felt how my eyes turned into organs of skin and touch through haptic looking (Marks, 2002, 2–3): They invited me to sense and to feel the movements as chills on my arms and legs and as small drops of salty water flowing slowly from my eyes to my cheek. Philosopher Marks (2002) uses the concept of haptic looking to highlight the connections between looking and other sensorial perceptions. Marks writes how the gaze moves actively over the surface of the screen while it blurs the things that are “seen,” rather than focuses which gives space for other sensorial perceptions in the processes of looking.

However, the sensation of bodily diversity I experienced in the situation of moving happened not in dual relations between me, and the screen; the main affection arose from the kinesthetic connections I felt to the other participants who were each dancing in their own boxes. The sensation that arose at the moment made me, in turn, aware of how the combination of multisensoriality and affective and kinesthetic connections manages to blur classifications related to different organs, body parts, and subjectivity (Smith and Roche, 2015; Piitulainen and Mekler, Accepted/In press) when it, for example, gave me the feeling of becoming more than one and intersubjectively one in the shared moment of moving together. These experiences of intersubjectivity and bodily diversity evoked a special sense of communality, making me momentarily feel less lonely and safe in times of social isolation. However, I also sensed something was missing. Feeling safe and connected existed parallel to the sensations of longing for physical touch and shared physical space. The tiny teardrop embodied both.

In my example, experiences of communality, affection, and multisensorial and intersubjective perceptions about my body enabled feelings of safety. Furthermore, the instability of corporal sensations evoked a certain feeling of freedom when classifications between the functionality of different senses and organs were deconstructed in the moment of moving and being moved. However, while these affective intra-actions and experiences of multisensoriality minimized the effects of loneliness, they were also challenged by the sensations of longing.

Performance scholar Mezur (2013) states, leaning on Marks (2002), how viewing as a multisensorial act in virtual communication enables people to distance themselves and simultaneously to come so close that the perceived things that are touchable, yet out of touch, blur and even disappear (Marks, 2002, p. 92). This sort of disappearance evokes certain feelings of longing. Mezur discusses sensations of longing through the experiences of performers and viewers of a Japanese interactive dance installation. She uses the expressions “cold pressure” and “cold burn” regarding the ambivalent feelings that these forms of touching evoke (Marks, 2002, p. 90).

The discussion on haptic looking as a moving, multisensorial act helps us open a dialogue about the instability of corporeal sensations in their broader connection to society and culture (Mezur, 2013, 90) during crises. Going beyond the normative understanding of body and touch matters to the feeling of safety, since it increases communality, and makes us feel accepted, empowered, and less lonely in a holistic unbiased way. However, the multiplicity of the body that can be experienced when our bodies intersect with technologies does not always come with only positive aspects. Sometimes, this multiplicity can evoke the opposite when authenticity is missing, which might affect one's feelings about their body, thus generating longings for physical interaction. The wall that exists in virtual communication between its participants cannot be ignored which makes an authentic interaction, where feelings of communality and physicality are transmitted and restored by technology challenging.

## 4 Ambivalences in feelings of safety and communality experienced through virtual/physical dance

The coexisting duality regarding feelings of safety in online dancing was also discussed in my interviews. One interviewee from Ostrobothnia, who suffered from psychosis a couple of years before the pandemic erupted, described how her experience of virtual/physical dancing was strongly related to her mental state.

Noora: How did you experience the collective online meetings with the AbilityDance organization?

Kirsi: Well, quite versatile. It was quite much related to my personal condition. I have had these panic attacks and anxiety so that sometimes it was very hard for me to be part distantly. The more anxious I feel, the harder it is for me to take part online. Because the interaction is different, so it is harder for me to be myself and to interact with others. [...] But then I have also experienced that sometimes it has been very lovely to be at least somehow able to interact with people [Ostrobothnia, 7/5/2021].

In the interview, Kirsi^3^, 38, discussed how the different forms of interaction made it harder for her to be herself. In that case, the idea of feeling safe relating to overcoming loneliness through affective intra-actions fails. Sinikka, 86, in turn, talked about the act of “breathing the same air in a live performance or a dance class” as a form of touch and contrasted it with virtual dance.

Sinikka: Being in the same physical space is already lived touch. Physical touch is closer, but it is a form of touch too when we are breathing the same air and seeing others and so on [7/21/2021].

During the pandemic, people in vulnerable positions, such as those with chronic illnesses, disabilities, and the elderly, were especially protected from the dangers of getting infected through physical touch or inhaling air in which the virus could exist. Sinikka's quote makes us look at the multiplicity of touch regarding intra-actions between different movements, bodies, multisensorial sensations, technology, and space, as well as the air we breathe. In her example, the different spaces in virtual communication build a barrier to experiencing touch via breathing the same air. Breathing the same air is associated with touch and communality, while it separated people during the pandemic for the sake of protection. This practice is irreplaceable in virtual forms of communication.

Gender Studies scholar Górska (2016) uses the terms corpomateriality^4^ and corpo-affectivity to reflect on how breathing is enacted in different affective processes of relating (Alaimo and Hekman, 2008, p. 238; Górska, 2016, p. 47, 49, 50). Moreover, breathing manages to “challenge internal and external boundaries of human corpomateriality” when the environment enters the body during inhalation (Górska, 2016, p. 50, 250). Górska discusses relationalities between affect and social power relations in breathing, shedding light on situations of social and political inequality concerning, for example, toxic inequalities and a breathable life (Górska, 2018, p. 251).

While breathing manages to complicate notions of the self, the other, and the environment (Górska, 2018, p. 250), touching as a multisensorial, affective contact (Brandstetter et al., 2013, p. 3) similarly enables the blurring of classifications between the one who is touched and the one who touches, creating, and affective co-becoming. In online dancing, we might experience how physical/virtual dance diversifies our understanding of the body, touch, and safety, harboring the intentions of marginalized communities to increase inclusiveness and the ability in society during crises. Conversely, these multiple forms of touching and being touched enact new modes of longing, making us aware of ambivalences regarding feelings of safety in connection to ideas about communality.

## 5 Discussion

This article discussed how the multisensorial ways dance manages to touch us and help us experience the feelings of safety through communality in the moment of moving together. I shed light on the role of affect in generating feelings of safety by using the concepts of haptic visuality and intra-action in the analysis of my material. The coexistence between multisensorial perceptions and sensations of longing, in turn, brought me to ambivalence regarding associations between feelings of safety and communality in virtual/physical dance. I discussed these ambivalences through the example of breathing the same air as a form of touch that separated people during the COVID-19 pandemic for the sake of protection.

Despite the ambivalences, I argue that artistic responses, whether virtual or physical and made under the intention of care, help us experience affective connections and contribute toward anti-normative understandings of the body and identity. These affective connections help us momentarily imagine a shared and better world. In an ideal case, the feelings of communality and inclusivity can turn into culturally shared emotions (Rinne et al., 2020, p. 8), generating modes of healing—individually but societally.

Furthermore, in post-pandemic times, it is important to contribute toward holistic experiences of healing and safety that are related to anti-normative perspectives about the body, the senses, and identity. After the pandemic, the gap between the privileged and the vulnerable has grown as, for example, far-right extremism and social and economic inequality increased worldwide. Hence, anti-normative, intersectional discussions about safety must be included in measuring safety, which might help us minimize some of the adverse effects a crisis such as COVID-19 will have on future inequalities and the experiences of the safety of the marginalized. The multisensorial forms of expression, for example, typical in inclusive dance, offer a fruitful basis to approach the body, the senses, and identity through anti-normativity beyond verbal classifications.

## Data availability statement

The raw data supporting the conclusions of this article will be made available by the authors, without undue reservation.

## Ethics statement

Written informed consent was obtained from the individual(s) for the publication of any potentially identifiable images or data included in this article.

## Author contributions

NO: Writing – original draft.

## Footnotes

^1^With the term virtual/physical dance I refer to the physical experiences made in the practices of online dancing.

^2^The terminology refers to dance practices with able-bodied and non-able-bodied participants.

^3^All the names, mentioned from the interviews are pseudo names. I translated the interviews that were held in Finish to English.

^4^Lykke used the concept corpomaterial for the first time to combine different feminist discussions on corporality and the materiality of bodies (Lykke, 2010, p. 107; Górska, 2018, p. 250).
